# FEASIBILITY OF CONTINUOUS PASSIVE MONITORING OF DAILY FUNCTION AND SYMPTOMS IN OLDER ADULTS WITH MULTIPLE MYELOMA

**DOI:** 10.1093/geroni/igad104.2816

**Published:** 2023-12-21

**Authors:** Deanne Tibbitts, Rebecca Silbermann, Eric Roeland, Jason Webb, Gabrielle Meyers, Jackilen Shannon, Jeffrey Kaye, Kerri Winters-Stone

**Affiliations:** Oregon Health & Science University, Portland, Oregon, United States; Oregon Health & Science University, Portland, Oregon, United States; Oregon Health & Science University, Portland, Oregon, United States; Oregon Health & Science University, Portland, Oregon, United States; Oregon Health & Science University, Portland, Oregon, United States; Oregon Health & Science University, Portland, Oregon, United States; Oregon Health & Science University, Portland, Oregon, United States; Oregon Health & Science University, Portland, Oregon, United States

## Abstract

The impact of cancer and cancer treatment on older adults’ day-to-day health and functioning remains poorly characterized. Therefore, we evaluated the feasibility of using home-based, continuous passive monitoring of daily life functioning plus symptom surveys in older adults with multiple myeloma (MM). We assessed accrual, uptake of passive monitoring, retention, survey completion, and data completeness. Passive monitoring included infrared motion sensors (activity), door contact sensors (time out-of-home), watch (activity/sleep), bed mat (sleep), biometric scale (weight), electronic pillbox (medication-taking habits), and computer-use software (computer habits). A weekly online survey assessed health events (i.e., falls, hospitalization/ED visits, pain) and treatment-related symptoms (PRO-CTCAE). We enrolled 20 patients with MM of 55 referred (36% accrual) with a median age of 71 years (35% female); 8 patients enrolled with a care partner. Half of patients agreed to passive monitoring and weekly surveys; half completed surveys only. Refusal of passive monitoring was primarily due to concerns about privacy/intrusiveness. Retention over 17 months was 85% (3 dropouts from the survey-only group). Survey completion was 82% (missing reasons included “poor health” and “too busy”) with a median completion time of 4.6 minutes. Data completeness for passive monitoring ranged from 66% for watch-based sleep (n=9 patients) to 93% for home-based activity (n=14 homes). In sum, passive monitoring of daily life functioning plus symptom surveys is feasible among older adults with MM. Future research will integrate daily functioning with symptom data to examine longitudinal variations in health and develop digital biomarkers to inform optimal timing of future interventional studies.

